# Barriers and enablers to reporting pregnancy and adverse pregnancy outcomes in population-based surveys: EN-INDEPTH study

**DOI:** 10.1186/s12963-020-00228-x

**Published:** 2021-02-08

**Authors:** Doris Kwesiga, Charlotte Tawiah, Md Ali Imam, Adane Kebede Tesega, Tryphena Nareeba, Yeetey A K Enuameh, Gashaw A. Biks, Grace Manu, Alexandra Beedle, Nafisa Delwar, Ane B. Fisker, Peter Waiswa, Joy E. Lawn, Hannah Blencowe, the Every Newborn-INDEPTH Study Collaborative Group

**Affiliations:** 1 Department of Health Policy, Planning and Management, Makerere University School of Public Health, Kampala, Uganda; 2 Centre of Excellence for Maternal Newborn and Child Health Research, Makerere University, Kampala, Uganda; 3 Department of Women and Children’s Health, Uppsala University, Uppsala, Sweden; 4 Kintampo Health Research Centre, Kintampo, Ghana; 5 Health Systems and Population Studies Division, International Centre for Diarrhoeal Disease Research, Dhaka, Bangladesh; 6 Dabat Research Centre Health and Demographic Surveillance System, Dabat, Ethiopia; 7 Department of Health Systems and Policy, University of Gondar Institute of Public Health, Gondar, Ethiopia; 8 IgangaMayuge Health and Demographic Surveillance System, Makerere University Centre for Health and Population Research, Makerere, Uganda; 9 Department of Epidemiology and Biostatistics, Kwame Nkrumah University of Science and Technology, Kumasi, Ghana; 10 Maternal, Adolescent, Reproductive & Child Health (MARCH) Centre, London School of Hygiene & Tropical Medicine, London, UK; 11 Bandim Health Project, Bissau, Guinea-Bissau; 12 Research Centre for Vitamins and Vaccines, Statens Serum Institut, Copenhagen, Denmark; 13 Open Patient data Explorative Network (OPEN), Uni. of Southern Denmark, Odense, Denmark; 14 Centre of Excellence for Maternal Newborn and Child Health Research, Makerere University, Kampala, Uganda; 15 Department of Public Health Sciences, Karolinska Institutet, Stockholm, Sweden

**Keywords:** Pregnancy reporting, Neonatal, Stillbirth, Miscarriage, Adverse pregnancy outcomes, Stigma

## Abstract

**Background:**

Risks of neonatal death, stillbirth and miscarriage are highest in low- and middle-income countries (LMICs), where data has most gaps and estimates rely on household surveys, dependent on women reporting these events. Underreporting of pregnancy and adverse pregnancy outcomes (APOs) is common, but few studies have investigated barriers to reporting these in LMICs. The EN-INDEPTH multi-country study applied qualitative approaches to explore barriers and enablers to reporting pregnancy and APOs in surveys, including individual, community, cultural and interview level factors.

**Methods:**

The study was conducted in five Health and Demographic Surveillance System sites in Guinea-Bissau, Ethiopia, Uganda, Bangladesh and Ghana. Using an interpretative paradigm and phenomenology methodology, 28 focus group discussions were conducted with 82 EN-INDEPTH survey interviewers and supervisors and 172 women between February and August 2018. Thematic analysis was guided by an a priori codebook.

**Results:**

Survey interview processes influenced reporting of pregnancy and APOs. Women found questions about APOs intrusive and of unclear relevance. Across all sites, sociocultural and spiritual beliefs were major barriers to women reporting pregnancy, due to fear that harm would come to their baby. We identified several factors affecting reporting of APOs including reluctance to speak about sad memories and variation in recognition of the baby’s value, especially for APOs at earlier gestation. Overlaps in local understanding and terminology for APOs may also contribute to misreporting, for example between miscarriages and stillbirths. Interviewers’ skills and training were the keys to enabling respondents to open up, as was privacy during interviews.

**Conclusion:**

Sociocultural beliefs and psycho-social impacts of APOs play a large part in underreporting these events. Interviewers’ skills, careful tool development and translation are the keys to obtaining accurate information. Reporting could be improved with clearer explanations of survey purpose and benefits to respondents and enhanced interviewer training on probing, building rapport and empathy.

## Key findings

**Table:** 

**What is new?**	
• **What was known already**: Many low- and middle-income countries rely on population-based surveys like the Demographic and Health Surveys to measure pregnancy and adverse pregnancy outcomes (APOs). However, there are challenges with their data quality, including misclassification and omission of events, as well as social norms that influence reporting of pregnancy and APOs. • **What was done:** 28 FGDs were conducted across five HDSS sites in five countries in sub-Saharan Africa and South Asia, involving 172 women and 82 survey interviewers (eight of these interviewers were supervisors from Matlab). Qualitative methods were used to explore barriers and enablers to reporting of pregnancy and multiple APOs, notably miscarriages, stillbirths and neonatal deaths.	
**What was found?**	
• **Barriers/enablers:** o **Methodological barriers to reporting pregnancies and APOs in surveys**: these were mainly generic, such as challenges with survey tools and consistency in training, but context-specific too, including local understanding of constructs. Interviewer skills and knowledge are critical in accurate collection of data. o **Sociocultural barriers to reporting pregnancy and APOs:** these were remarkably similar across five different settings, especially religious and cultural beliefs and stigma. There are also women-specific barriers, notably for adolescents and younger women. o **Psycho-social impact of APOs:** grief associated with loss means that many mothers do not want to recount these negative experiences, especially for a purpose they do not understand. • **Differences in reporting APOs** o **Variation in severity of reporting barriers by APO:** The results suggest that there is a “dose response,” with higher barriers to reporting APOs at earlier gestations and those with more attached stigma, notably miscarriage, then stillbirth, with neonatal deaths more likely to be reported but still less likely than older child deaths. This is evident in the various burial and mourning practices.	
**What next in measurement and research?**	
• **Measurement improvement now:** o **Tools and local adaption:** ensure translations of key terms are culturally and linguistically accurate and grounded in the local cultural context. o **Interviewer soft skills:** develop skills in rapport building, probing and empathy among survey interviewers through enhanced training with interactive and reciprocal exchanges. o **Survey purpose and use of data**: provide interviewers with adequate knowledge about the survey, and ensure this is well communicated to respondents, especially its benefits to their health and that of the broader community, with confidentiality emphasised. • **Research needed:** o **Contextual adaptation guide:** research is needed on how to improve tools for surveys on pregnancy and APOs, to ensure more accurate and consistent reporting in different cultures and languages. o **Enhanced training module for interviewers:** there is a need for development and evaluation of enhanced training materials on pregnancy and APOs to be included in survey fieldworker training, with prospective assessment to understand the effect of this enhanced training.	

## Background

Adverse pregnancy outcomes (APOs), including miscarriages, stillbirths and neonatal deaths, are a major burden associated with long-term psychological and social effects [1]. In 2017, an estimated 2.5 million neonatal deaths occurred globally [2] and a further 2.6 million stillbirths (deaths in the last 3 months of pregnancy or during childbirth) [3]. Around 11–22% of known pregnancies end in miscarriage, most of these in the first trimester [4]. These APOs are often underreported and can negatively affect maternal health as well as that of fathers and families, leading to grief, depression and social withdrawal [5–8].

The risk of these APOs is highest in sub-Saharan Africa (SSA) and South Asia, yet these highest burden countries have the most gaps in their civil registration and vital statistics (CRVS) and data systems—the inverse data law [9]. Inaccurate data can lead to underestimation in national and local statistics used for planning and priority setting [9] and invisibility in data and society can lead to under-investment. Reliable trends are important for monitoring progress to achieving the Sustainable Development Goals (SDGs) and tracking inequalities, including Every Newborn Action Plan (ENAP) targets to reduce preventable newborn deaths and stillbirths [10, 11]. The ENAP aims to end preventable newborn deaths and stillbirths, and by 2030 to reduce neonatal deaths to 12 or fewer per 1000 live births and stillbirths to 12 or fewer per 1000 total births in every country [10].

Many low- and middle-income countries (LMICs) rely on nationally representative population-based household surveys such as the Demographic and Health Surveys (DHS) and Multiple Indicator Cluster Surveys (MICS) to measure pregnancies and APOs, and surveys are the main data source for high-burden countries, accounting for 75% of the global burden of APOs [12, 13]. These surveys are conducted approximately every 5 years in more than 93 countries. However, there are challenges associated with survey data. General data quality can be affected by language and translation, timing of interview and other factors [13]. Survey data including those on APOs face further challenges, with omission and misclassification of events common [14, 15].

Few studies have sought to understand barriers specific to reporting pregnancy and APOs in surveys. A study in Tanzania including mothers and female elders found that maternal report of pregnancy loss was influenced by social norms, including silence around discussing the loss [16]. They also identified terminological and methodological challenges to reporting. Similar findings were reported in a study in Afghanistan which used in-depth interviews [17]. However, these studies focused on single countries and did not include interviewers’ perspectives.

The Every Newborn-International Network for the Demographic Evaluation of Populations and their Health (EN-INDEPTH) study was undertaken in five Health and Demographic Surveillance System (HDSS) sites, with the overall aim of informing improvements in the measurement of pregnancy outcomes in population-based household surveys. Details of the study protocol and results of the primary objective to randomly compare two methods of retrospective recording of pregnancy outcomes (a full birth history with additional questions on pregnancy losses, as per the current standard in phase 7 of DHS, and a full pregnancy history) are published elsewhere [18, 19].

This paper is part of a series of papers from the EN-INDEPTH study. Our aim was to use comparable tools and methods across five health and demographic surveillance system sites to:

1. ***Describe barriers and enablers to women’s reporting of pregnancy and APOs***, including individual, community, cultural and interview level factors.
2. ***Inform measurement improvements*** in population-based surveys.

## Methods

### Study design

**Fig. 1:**
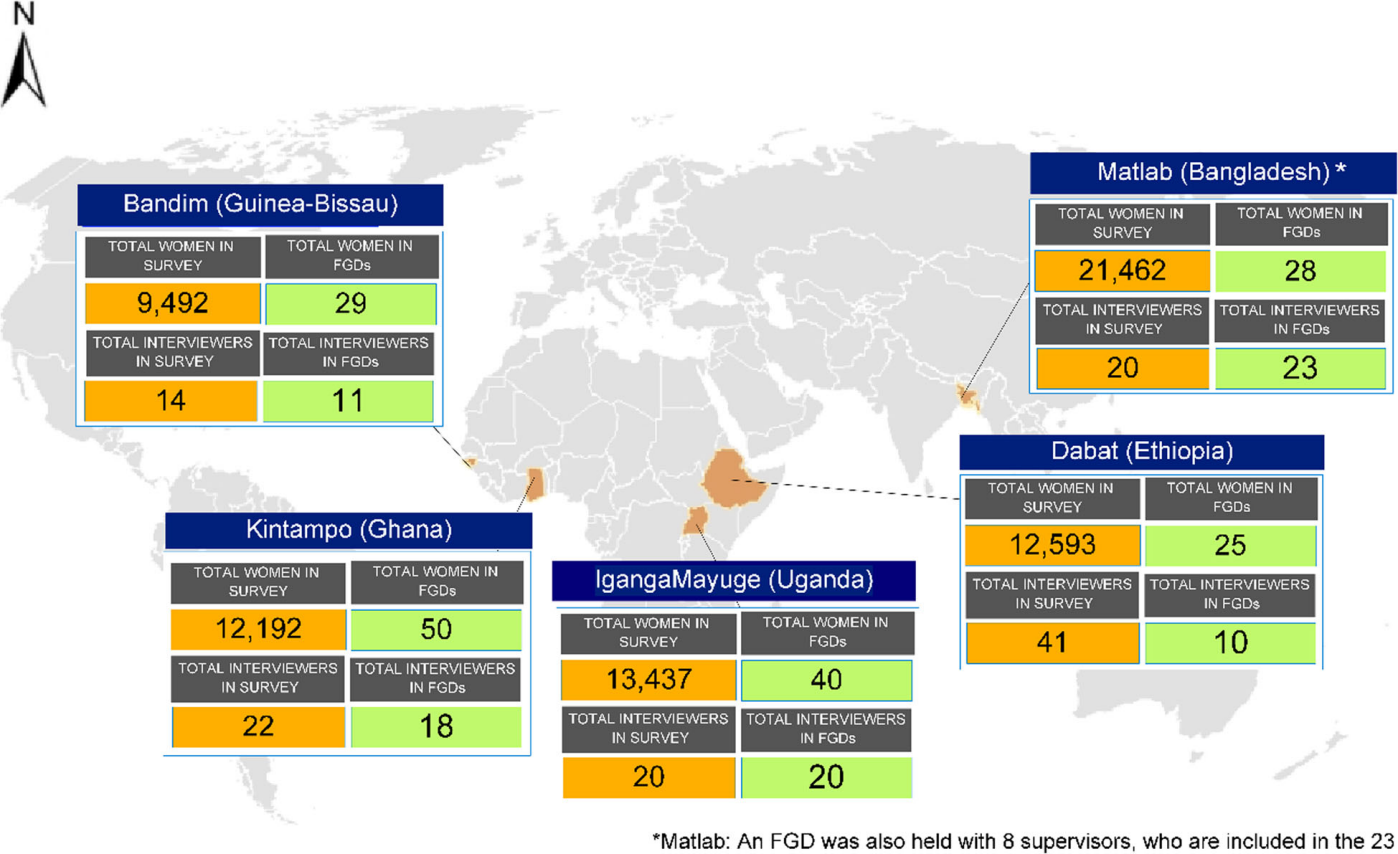
Location and total number of respondents in the EN-INDEPTH survey and interviews

The EN-INDEPTH study was undertaken in five HDSS sites which were part of the INDEPTH network: Bandim in Guinea Bissau, Dabat in Ethiopia, IgangaMayuge in Uganda, Matlab in Bangladesh and Kintampo in Ghana. A cross-sectional population-based survey of 69,176 women of reproductive age was undertaken between July 2017 and August 2018. Focus group discussions (FGDs) with women (survey respondents) and survey interviewers (and also supervisors from Matlab) were undertaken to understand the common social norms and practices around reporting pregnancy and APOs between February and August 2018 (Fig. 1). One FGD was conducted with supervisors from Matlab, which was undertaken to explore any differences between their perspective and those of the interviewers on the ground.

### Participant selection

Women were purposively selected from the pool of respondents who had participated in the EN-INDEPTH survey to ensure diversity by age, place of residence (urban/rural) and experience of APOs. Interviewers were purposively selected from the EN-INDEPTH survey interviewers and included both females and males where possible.

While all sites recruited participants face-to-face, telephone calls were also made as part of recruitment in Matlab and IgangaMayuge, and written information was also left in Bandim if the respondent was not present. In Bandim, four out of 24 women approached in the rural areas and 19 out of 30 approached in the urban areas did not come for the FGDs. There were no refusals or dropouts in the other four sites.

### Data collection

FGD guides were developed by a multi-country qualitative working group. The women’s FGD guide examined experiences with the EN-INDEPTH survey, how they and others disclose pregnancy and APOs, perceptions on gestational age and birth weight and knowledge and practices around pregnancy and APOs. The interviewers' guide considered experiences with the survey process and collecting data on pregnancy and APOs (Additional file 1). The DHS interviewer training manual informed the development of a standard FGD training manual, which was used to train moderators and note-takers in all sites except Bandim. FGD guides were translated into the local language.

FGDs were held in accessible places, including within the community (under trees, sheds, courtyards), at nearby health facilities, at the HDSS offices, and for some EN-INDEPTH interviewers, at their workplace. Efforts to ensure privacy were made, with only respondents and researchers present. FGDs typically lasted between 1.5 and 2 h, and no repeat FGDs were conducted. Notes from FGDs were written and discussions recorded using a tablet or tape recorder, in addition to field notes. Upon completion, the team transcribed the FGDs in the survey language using a combination of notes and audio recordings. These were then translated into English but were not returned to participants for comments.

### Research team and reflexivity

In all sites, the interview teams were led by researchers with either Master’s or PhD degrees, who jointly formed a collaborative, consistent, multi-country qualitative working group. While the FGDs were organised by the HDSS teams, which are known within the local communities, there was no direct personal relationship between researchers and respondents. All those involved in data collection including moderators and note-takers were nationals of the respective country. In three HDSS sites, work was led by a staff member with experience in conducting qualitative research. HDSS staff in each site internally recruited experienced FGD moderators and note takers with fluency in the most commonly spoken language, apart from Bandim where they were externally recruited. In Bandim alone, data analysis was undertaken by non-Bissau-Guinean researchers with support in understanding the local context from the FGD moderator and the Bandim HDSS team. The lead researchers were not moderators or note takers, although they attended the FGDs except in Bandim. In IgangaMayuge and Dabat, moderators were males, with females in Matlab and Kintampo, while Bandim had both sexes. Note takers were female in Matlab and Bandim, while Kintampo, IgangaMayuge and Dabat had male note takers.

### Data analysis

This study used an interpretative paradigm which aims to understand the ways in which people behave, what things mean to them and how they interpret the world and phenomenology methodology to seek to understand peoples’ “lived” experiences [20]. A data management and analysis plan was jointly developed by the multi-site qualitative working group and used across all sites. Both inductive and deductive coding were used. Thematic analysis of the English transcripts was conducted in NViVo version 12 using an iterative process guided by an a priori codebook based on the research teams’ experiences and published literature [16, 21, 22]. Additionally, new codes were included for themes identified during analysis.

Coding for each site was done independently by two coders who met regularly face-to-face and online to discuss identified codes and themes. These coders were the lead researchers for the work in each site, except Bandim where analysis was led by DK, the overall lead researcher for this qualitative study and AB who was externally recruited. The multi-site qualitative working group (most of whom were involved in coding) had conference calls to discuss and synthesise findings, culminating in a face-to-face analysis meeting in February 2019. In these discussions, teams were able to compare coding and agree on the identification of themes. Data saturation was discussed and noted by each analysis team in the different sites, and COREQ guidelines have been used in this paper [23] (Additional file 2).

## Results

### Overall

A total of 28 FGDs were conducted between February and August 2018, of which 19 were conducted with women (*n* = 172) and nine with survey interviewers (*n* = 82). The demographic characteristics of interviewers from Matlab included eight supervisors, who had their own separate FGD; no additional themes were derived from this FGD. Additional file 3 provides background information on the HDSS sites and Additional files 4A and 4B a summary of respondents’ demographic details.

Multiple constructs and challenges which influence reporting of pregnancy and APOs were identified (Table 1). These are further discussed below, including verbatim quotations highlighting responses of both women and interviewers.

**Table 1:** Overview of themes: barriers to reporting of pregnancy and adverse pregnancy outcomes (APOs)

Major theme	Sub-theme	Barriers to reporting of pregnancy	Barriers to reporting of adverse outcomes
**Socio-cultural and spiritual beliefs**	Stigma	Unplanned pregnancy, fear of judgement	Blame for women for these APOs; fear of judgement and stigma from the community
	Religion	Religion discourages pregnancy before marriage	Fatalism about APOs or seen as punishment
	Witchcraft and spiritual beliefs	People with ill intentions will harm the baby; evil spirits attracted by disclosure	Miscarriage and stillbirth caused by spiritual harm, or punishment; talking about it may cause a reoccurrence of APO
	Variation in recognition of the baby’s value		Baby not considered human; value attached to a baby influences reaction to death
	Burial and mourning practices		Often different for APOs, especially miscarriages and stillbirths; secretive burials
	Descriptions/names of APOS		Names with negative meanings; same names used to mean different APOs
	Trust/privacy	Lack of trust, unsure of privacy and confidentiality of their information	
	Gender & patriarchy	Men who do not want their wives to be interviewed; more barriers for interviewer of a particular sex	
**Woman-specific**	Age	Adolescent girls: secretive, scared and shy	
	Individual response to pregnancy	Woman unsure about pregnancy and considering if to terminate the pregnancy	
**Psycho-social impact of APOs**	Negative psychological and emotional impact		APOs cause grief and sadness. Talking about them resurrects bad memories
**Survey interview tools and processes**	Specific to these outcomes	Questions on APOs considered irrelevant, purpose and benefits not clear to women Questions are intrusive on a sensitive topic	
	General	Interviewer skills, strategies and knowledge Long interview tools with apparently repetitive questions Physical distance challenges in locating respondents High workload for interviewers Inconvenient time of interviews Multiple call-backs to a household to locate right respondent	

Note: Enablers to reporting of pregnancy and adverse pregnancy outcomes were generally the converse of the barriers. Specific enablers mentioned focused on the ‘Survey interview tools and processes’ theme and included enabling factors around the interview process, interviewer skills and strategies and the respondents understanding of and perceived benefits from the interview

### Barriers related to interview tools and processes

A number of women viewed questions about pregnancy as irrelevant. They did not understand why data collectors were interested in their pregnancy or how women would benefit from revealing information. Furthermore, across all sites, the lengthy and repetitive consent form and survey tools reportedly tired out both interviewers and respondents, resulting in loss of concentration and interest among respondents. Interviewers reported that women were more likely to provide any answer, even if incorrect, to end the interview quickly.

Interviewers faced general process-related challenges. For instance, inconveniently timed interviews resulted in impatience on the respondents’ part and a shorter interview, affecting the quality of data. Furthermore, making multiple household callbacks to be able to conduct the interview was challenging especially in towns, where people went to work early, returned late and were generally less welcoming to interviewers, as reported in Kintampo and IgangaMayuge. Often, terrain and distance were challenging, and some interviewers reported having to follow a respondent to their farm and then return to locate the next households.

*Sometimes when you go to the house, some of them will be in a hurry to go to work so they sometimes do not give us the right responses… (Interviewer, Kintampo, Ghana)*

### Barriers to reporting of pregnancy

#### Socio-cultural and spiritual beliefs

In all sites, women feared stigma and judgement from the community if they disclosed their pregnancy. For instance, they feared being the subject of gossip if they already had young or many children, or if their youngest child had already grown up. Unmarried women and adolescent girls frequently hid their pregnancy due to stigma. Many women explained not wanting to disclose their pregnancy because if they later lost the pregnancy, they would be accused of lying or of having had an induced abortion. This also reduced the likelihood of reporting early pregnancy in the surveys.

*The problem may arise from some individuals who may label and say she gave birth while the previous child is an infant or she didn’t feed well the already born children but still she is getting another pregnancy. Or they may talk about whether you got the pregnancy from an unknown partner and this may bring another label to you, that the people may say that pregnancy (newborn) is called ‘diqala’ or ‘wofzerash’ meaning unknown source or from unknown father, which is very taboo and outlawed. Due to this and other social criticism we preferred to hide our pregnancy (Woman, Dabat, Ethiopia)*

*If she is pregnant for the first time, this is something that she was not expecting… you have this shame, this fear. For example, a woman like me who is not married to be pregnant, I am going to be embarrassed to tell people, for my colleagues to see me. Maybe those that have already been pregnant are not going to be embarrassed, but those that have never been pregnant before, I am going to be embarrassed to tell my boyfriend, I am going to say I have never been pregnant… (Woman, Bandim, Guinea Bissau)*

Religion was sometimes reported as detrimental to reporting pregnancy and as a source of stigma. In all sites, when a woman was unmarried and her religion discouraged pregnancy before marriage she would hold back from disclosing.

The role of witchcraft and spiritual beliefs, coupled with cultural beliefs, perceptions and practices, was predominant in all sites. Common perceptions included that people who were jealous or had ill intentions could harm the baby through witchcraft, for example by causing “*the stomach to disappear*”, or “*the pregnancy will not last*”, or “*evil eyes*”. In Matlab, miscarriage and stillbirth were specifically attributed to spiritual harm. To avoid this, women in all sites failed to disclose early pregnancy or hid until it showed itself or until delivery. Additionally, some women believed there was a high chance of an APO if they disclosed their pregnancy early, even without witchcraft and the fear was worse among women who previously had an APO.

*Culturally, it’s not good to tell everyone about the pregnancy. When you tell one about your pregnancy age, culturally they can take your footstep soil and bewitch you and you get a miscarriage, have caesarean birth or you may die during labour process. Therefore, it is better to keep silent and they just see (Woman, IgangaMayuge, Uganda)*

In all sites, lack of trust and confidentiality of information was a hindrance to disclosing pregnancy. Pregnancy was not usually disclosed before 3 months, except to close family and friends.

Gender and patriarchy sometimes presented barriers to reporting. Although not common, occasionally in IgangaMayuge and Bandim, men were reported as a hindrance, for instance, if they did not want their wives to be interviewed. Amongst some ethnic groups in Bandim, men were considered gatekeepers and often data collectors had to seek their consent and explain what the survey was about before speaking to the women.

*Sometimes when we arrive, the woman says her husband does not authorize her to speak with us (Interviewer, Bandim, Guinea-Bissau*)

#### Woman-specific factors

Issues such as a woman’s age and their intentions towards the pregnancy affected reporting in many of the sites. Data collectors noted that adolescent girls were often shy about issues to do with periods, pregnancy and sexual and reproductive health, limiting collection of accurate information. Furthermore, some of them still lived with their parents and were scared to reveal a pregnancy due to expected repercussions. Other women, as noted in Dabat, Kintampo and IgangaMayuge, hid their pregnancy because they intended to terminate it, particularly married or younger women. In other cases, non-disclosure was because the father of the baby had not yet accepted responsibility or the woman was worried that he would encourage her to abort it.

…*Also a woman will not tell you she is pregnant if she has not decided on whether to keep the pregnancy or not. So generally capturing early pregnancies is difficult (Interviewer, Kintampo, Ghana).*

### Barriers to reporting of adverse pregnancy outcomes

All barriers to reporting pregnancy also applied to reporting of APOs. For instance, concerning interview processes, women in all sites did not understand why interviewers asked about APOs, or of what benefit the information would be to either interviewer or the woman herself and found the questions intrusive.

*…but musawo [doctor] you have asked a number of times but now look at such questions. The children died and you won’t bring them back. Just ask for the ones who are still alive but the dead, miscarriages, stillbirths, how are you going to help us? It is useless and just time wasting to ask those questions (Interviewer, IgangaMayuge, Uganda)*

In Kintampo, it was thought that discussing an APO would cause the baby to “come back and worry you,” for instance as a stillbirth. There appeared to be an attitude of fatalism and acceptance of APOs among respondents and their communities. Indeed, most were aware of APOs happening around them or had experienced them. Respondents in all sites explained that often this is considered fate or God’s will.

Barriers that were specific to reporting of APOs only but not to reporting of pregnancy include the perceived value of the baby’s life and the descriptions and psycho-social impact of APOs, which we discuss below.

#### Variation in recognition of the baby’s value

The value placed on a baby’s life and how that varied with gestational age, or if born alive, influenced reporting of APOs in all sites both by women describing their personal experiences with APOs and by women and interviewers speaking more generally about societal attitudes. Where a woman or society felt that the baby was not yet a human, neither saw the importance in reporting its loss. Although more value appeared to be placed on a stillbirth since the woman had carried it for a longer time, respondents still mentioned these as not fully human and therefore not easily disclosed. Deaths of babies who had lived for a few days were considered the losses that would most affect the women and were viewed as babies whose existence and loss were more likely to be reported.

*One child of mine has been miscarried. I have seen, it was like a piece of meat. So, what was [there] to love about that piece of meat? (Woman, Matlab, Bangladesh)*

*With regard to a miscarriage, it’s not yet developed into a human and you don’t see the face but the one I have given birth to and have seen the face and cared for, when he/she dies it will pain me more than the miscarriage. It could be that the miscarriage didn’t even last for three or four months compared to the one I will carry for nine months, care and breastfeed. So I will value him or her more than the miscarriage (Woman, Kintampo, Ghana).*

These different perceptions in value of the baby were further evident in the various burial and mourning practices exhibited in different societies after an APO, as described by the women respondents. For instance, across all sites, miscarriages were not given a burial. In Matlab, findings show that many women do bury the piece of blood/meat miscarried under the ground, but this is to hide it, rather than formal burial. In most sites, burials for stillbirths and neonatal deaths reportedly occurred almost secretly or were carried out by a particular sex (women or men only). Sometimes, babies were not buried in coffins like adults but were wrapped in a cloth or put in a box and placed in a grave away from the main family burial grounds or at the back of the house. These practices indicated the need for silence around the APO, potentially influencing reporting of its occurrence. Contrastingly, in Matlab, there were only slight differences for burial of stillbirths compared to live born babies. Furthermore, the same religious and cultural rituals were used for newborn and adult burials. These include use of the burial shroud, “*janazah*” (funeral prayer), bathing the dead body, arranging “*milad*” (group prayer for the dead, usually conducted by the religious leaders), naming the baby and recitation of Quran.

#### Descriptions/names of adverse pregnancy outcome

Respondents were asked for the names of various APOs in their local languages. These names have predominantly negative connotations. In IgangaMayuge, a stillbirth or miscarriage is called “*empunha*” (useless) or “*ekintu*” (thing). In Dabat, the common name for a woman who has a stillbirth is “*woldo gedel*” in Amharic (deliverer and killer of her baby), while miscarriage is “*worja*” (pregnancy terminated unintentionally). In Kintampo, stillbirth is referred to as *“w’awo atwene*” (the woman has given birth and thrown it away). Miscarriages are referred to as “*w’apon ayinsen*” or “*ayinsen no aseɛ*” (the pregnancy is finished or spoilt), while in Bandim, one respondent defined it as “*Auor*” (give birth to but not to have). In Matlab, miscarriage was referred to as “*Nosto hoye geche*” (*fetus* terminated unintentionally), stillbirth as “*mora baccha hoiche*” or “*mrito baccha hoiche*” (baby has been born dead).

There was also some confusion amongst women in these communities in the terms distinguishing stillbirth and miscarriage. At times during the FGDs, it was difficult to know which APO was being referred to.

#### Psycho-social impact of APOs

A major barrier reported was the emotional and psychological impact of APOs. The loss of a baby was painful. Women reported not wanting to remember or share this painful experience and being asked directly about it revived sad memories. Interviewers described many instances where women could not talk due to grief, often becoming emotional, crying during the interview or saying they did not remember details. Similarly, women sometimes reported hiding APOs during surveys because recollection was too painful.

*As I have told you before, acquiring information is difficult on adverse pregnancy outcomes. Talking about the dead child is uncommon in the community. It is worse when it is neonatal death or when children get older as compared to the miscarriages, abortion and stillbirth because they remember the characteristics that they have seen. Therefore, women will be even tearing when you talk about a newly lost newborn. This makes the data collection difficult in the case of neonatal deaths (Interviewer, Dabat, Ethiopia)*

### Enablers to reporting of pregnancy and APOs

#### Interview process

The privacy of the interview setting and assurance of confidentiality encouraged women to open up. Women reported confidently sharing information with interviewers who would not reveal it to others. Interviewers highlighted that the interview language used was instrumental in obtaining accurate information, particularly when both interviewer and respondent were able to communicate in the same language.

The convenience of interview timing was a major facilitator to easing reporting in all sites. Both women and data collectors reported that when a woman had finished many of her domestic chores, she was more agreeable to answering questions. Data collectors highlighted the value of a good and clear consenting process at the start of the interview and thought that women were more prepared to sit through interviews, including the more upsetting questions about APOs when they had a clear description of what the survey’s purpose was.

*We conduct a one-on-one interview and there is privacy, so if you read out the informed consent and the person is told why you have visited her, the woman will be sure of confidentiality since the consent brings out all that message. It makes them free and gives us the information… (Interviewer, IgangaMayuge, Uganda)*

#### Interviewer skills and strategies

Building good rapport from the start of the interview, friendliness and earning the woman’s trust encouraged women to share their experiences. Interviewers’ understanding of and respect for local culture was important as part of their community or household entry strategy, for instance, seeking men’s consent first where required. They also avoided exhibiting arrogance or disdain on arrival at the respondent’s home. Some of these skills they mentioned learning from survey training but others were from previous field experience.

Across all sites, interviewers used empathy and sensitivity to obtain information on APOs. They often pretended to have had a similar experience which they shared, in order to make the woman comfortable enough to confide in them, which worked well. They also displayed patience and sympathy, particularly while discussing APOs. While waiting for the woman to regain her composure, interviewers sometimes asked another set of questions in the tool and later returned to the APOs. Additionally, good probing skills helped them to follow-up and to get respondents to open up.

*In my perspective the question about the abortion, stillbirth or dead infant may be very important to link the causes of death. But the way of asking such sensitive questions must take a friendly approach and care must be taken not to disappoint the women who have suffered. If you approach the woman kindly, share condolence and give her time to talk about her worries you can get the right information and these women will be treated well and they will give credit to you. The problem is most data collectors are very speedy and without conscious understanding of the women’s grief they started to ask directly about this sensitive issue. Consequently, we end up with the wrong information or a quarrel may be raised (Woman, Dabat, Ethiopia).*

#### Respondents’ understanding and perceived benefits from the interview

The frequent belief that interviewers were part of the HDSS and were therefore health workers often made women more responsive to questions. Women also asked them about different aspects of health and viewed the survey as a good learning opportunity. It was reported that once women knew and understood the benefit of the information they were giving and could link it to their health or that of their children, they expressed eagerness to participate.

## Discussion

### Discussion of findings

In this paper, we report various barriers and enablers to reporting pregnancy and APOs, including the interview tools and processes, socio-cultural and spiritual beliefs, woman-specific factors, the value placed on a baby’s life and the terminology and psycho-social impact of APOs. Overall, both women and interviewers across all the five study sites described similar barriers and enablers including the role of socio-cultural and spiritual factors and challenges around interview tools and processes. Women provided more contextual information on socio-cultural aspects, notably fear of harm to them or their baby if they disclosed the pregnancy, including stigma and judgement. They also expressed dissatisfaction with questions that seemed intrusive or irrelevant, without clear rationale or benefit to them or their community. We were surprised at the consistency of the barriers across five very varied contexts, as we had expected more context-specific variation. Many barriers we identified are consistent with existing literature [16, 21, 22, 24, 25].

Interviewers provided deeper insights on challenges in survey operationalisation, for instance locating and approaching appropriate respondents and the consent process. They highlighted various facilitators to reporting pregnancy and APOs. The interviewers were able to provide firsthand information about the extent of the psycho-social impact of APOs according to their observations.

Methodological barriers to reporting pregnancies and APOs in surveys were mainly generic, such as challenges with survey tools and practicalities of implementation. However, there were some context-specific barriers, for example local understanding of constructs used. Interviewer skills were instrumental in enabling collection of data on pregnancy and APOs. This is similar to a Mali study, where interviewers trained to be empathetic and understanding were more likely to gain the respondent’s trust, leading to more truthful reporting [26]. Furthermore, a household survey to determine the burden of APOs in Uganda noted that higher reporting of stillbirths could be due to women’s trust in interviewers [27]. However, they did not explore more on circumstances needed for women to reveal APOs. Influence of the interviewer role has been identified elsewhere, including in reporting sexual behaviour during surveys among adolescent girls in Kenya [28].

Sociocultural barriers to reporting of pregnancy and APOs were extraordinarily similar across the five different settings. This was especially so for stigma, witchcraft, religion and spiritual beliefs. Studies in South Africa, Ethiopia, Gambia, Uganda and Cameroon identified similar barriers to reporting APOs, as well as grief and patriarchy [6, 21, 22, 25, 29–34]. Consistently across all sites in our study, cultural factors played a role in encouraging silence around pregnancy and APOs. Indeed, pregnancy in Africa has been noted as a private event that is initially not revealed to the whole community [35, 36]. Women are often silent about it except to a few close people and therefore unlikely to disclose early pregnancies to interviewers [25]. Similar to our study, in Gambia, this secrecy was to avoid gossip or to prevent evil spirits that may be sent by ill-intentioned people to harm the pregnancy [29].

Our study found women-specific barriers, notably for adolescents and younger women where obtaining information was perceived as challenging due to their vulnerability, to fear of repercussions of getting pregnant, not knowing whom to confide in and shyness, as also found in the Gambian study [29]. Evidence from studies in other fields, for instance, reporting gender-based violence in surveys have identified underreporting, with the woman’s age given among the reasons [37].

Consistent with most previous studies, we also found fear of judgement and stigma after an APO. In the UK, women who had stillbirth experienced stigma that resulted in loss of identity as a mother, breakdown of social networks and relationships with one’s partner [38]. In rural Vietnam, fear of stigma was a barrier to the quality and accuracy of reporting deaths [31]. However, in Afghanistan, it was reported that stillbirths were openly discussed in communities and rarely stigmatised unless they had congenital abnormalities; rather, it was said that talking about a stillbirth could prevent it happening again [17]. Challenges with stigma have been cited as influencing reporting in other studies beyond pregnancy and APOs. For instance, a study using DHS data to analyse underreporting of gender-based violence (GBV) identified embarrassment, normalisation of violence and not seeing the importance of reporting as reasons for not reporting GBV [37]. In Pakistan, underreporting of gender-based violence was partly due to not wanting to “dishonor” ones family and cynicism about support from leaders once a case was reported [39].

An important and novel finding is that of an apparent “dose response”, with more barriers to reporting APOs occurring at earlier gestations, in part related to the lower societal recognition of them in terms of mourning or recognition of loss and also the associated stigma. Therefore, miscarriage is less likely to be reported than other APOs. Stillbirths are also often less likely to be reported than neonatal deaths and neonatal deaths less reported than older child deaths. Part of this dose-response relationship is evident in the various burial and mourning practices and the perceptions of value for the different APOs. Given these findings of more stigma associated with losing a baby at a younger gestation, it is possible that whilst women might not report these events at all, when they are reported they may overestimate gestational age. Where pregnancy events are reported in surveys, interviewers should be trained to facilitate as accurate recording of gestational age as possible, for example using hand-held antenatal care records where available [40].

The definitions of APOs, the negative connotations they carry and the confusion around what exactly is being referred to as shown in this study are a cause for concern with regard to reporting. In Tanzania, reproductive narratives with women who had an APO and interviews with other females in the community showed that local language definitions of different APOs overlapped in meaning and were hard to differentiate; thus, women often reported having one kind of APO while describing another [16]. This could potentially result in misclassification of the event during reporting, with for instance a miscarriage reported as a stillbirth or vice versa, leading to underreporting of certain events. Indeed, confusion still exists globally especially over stillbirth definitions, even within the same country [35, 41]. More so, biomedical definitions of APOs in Tanzania and Uganda were found to differ from local language definitions, resulting in confusion [16, 21]. It is possible that names that potentially blame the mother for the baby’s death or carry an element of shame are potentially likely to increase stigma around APOs, thus silence and underreporting or intentional misreporting of one APO as another.

The view on when exactly a fetus is considered a human being influences the “value” placed on them, associated mourning and burial processes of APOs and the likelihood of reporting. This value of personhood has long been a subject of global debate [25, 42, 43]. Among Pakistani Muslims in Britain, fetal personhood is bestowed with the “*azan”* and naming ceremonies, even if the baby dies shortly after that [43]. In Matlab, we found that the performance of religious rituals was almost the same for neonatal and adult deaths, with only slight differences for stillbirths. Similarly in an Afghanistan study, funerals were held and sometimes people prayed when babies were stillborn, despite this being frowned upon [17]. This was different from other sites in our study and literature from South Africa [25] and Ethiopia [22, 24], where burial and mourning practices were different or discouraged for APOs, especially miscarriages and stillbirths.

The multiple negative psycho-social impacts of APOs on women who experience these adverse outcomes have been highlighted in this and other studies [44]. Effects include social withdrawal, depression, somatic symptoms, anger, exhaustion, self-blame and breakdown of relationships [6, 44–46]. A review of literature on grief after miscarriage reported that while extreme grief declines and almost ends in 6 months, mourning continues through different stages of grief [6]. A grieving person may not be able, willing or expected to talk about their loss when asked about it during a survey. Other emotions include jealousy towards other pregnant women or those with children, social withdrawal, sadness, pain and fear among both parents [34]. The psycho-social impact of pregnancy and APOs on maternal mental health within low-income settings has not been widely studied, including understanding whether reporting of these in a survey adds to the trauma experienced by the women and if additional support or counselling should be linked to such surveys.

Given the dependence of the highest burden countries on household survey data for estimates of APOs, it is important for survey design to understand the multiple barriers to reporting. We propose potential solutions to address these, including ensuring the privacy of interviews, concise non-repetitive survey tools and culturally and linguistically accurate translations of key APOs terms when used across settings (Table 2). Furthermore, they should be grounded in the local cultural context.

**Table 2:** Potential solutions to improve reporting of pregnancy and adverse pregnancy outcomes in surveys

Area	Potential solutions
Interview process	Guaranteeing privacy and confidentiality
	Detailed and comprehensive consent
	Using language respondent is comfortable with and the interviewer is fluent in (most commonly used language)
	Realistic number of interviews per interviewer each day
Interviewer training	Development of enhanced training module for interviewers, with prospective assessment to understand its effect
	Interactive and reciprocal training that involves the experienced interviewers sharing their strategies to make respondents more comfortable and open up
	Classroom and field practice interviews
	Thorough training of interviewers to ensure in-depth understanding of the study and ability to explain its purpose to others
Interviewer skills, strategies and knowledge	Interviewer sensitivity to cultural semantics and taboos
	Building rapport
	Probing skills
	Empathy
	Sensitivity
	Understanding of psycho-social impact of grief
	Skills in interviewing adolescents
	Selecting interviewers from the same jurisdiction (though not the actual villages in which they will work)
Tools	Translation of tools using accurate and culturally recognised definitions e.g. of the different APOs
	Contextual adaptation guide to ensure more accurate and consistent reporting in different cultures and languages
	Shorter tools
Respondents and community	Conducting sensitisation before the study begins, including a clear explanation on purpose, benefits and any incentives to be given
	Consent from different gate keepers
	Provision of feedback on the study

Survey interviewers require soft skills in rapport building, often not emphasised in survey training, as well as probing, empathy and sensitivity to loss and intricacies of interviewing adolescents. These skills will improve the interaction between interviewer and respondent and quality of data. We suggest that interactive methods with reciprocal and more practical learning are important during training to address known barriers, including the understanding of the psycho-social impact of APOs.

The survey purpose and use of data are shown to be important both to the respondent and the interviewers. This includes providing interviewers with adequate knowledge about the survey and skills to communicate this to respondents, especially potential benefits to their and the broader community’s health. Interviewers must assure respondents of the confidentiality of their information to facilitate disclosure of APOs.

However, key gaps remain to improve APO reporting in household surveys. Research is needed on how to improve survey tools to ensure more accurate and consistent reporting of pregnancy and APOs in different cultures and languages, with results feeding into a contextual adaptation guide that could be used by DHS and others. The possible effect of differences in training on subsequent data and reporting is not yet well understood. Development and robust evaluation of an enhanced APO training module to be included in standardised interviewer training, which addresses known barriers, will be critical in maximising comparability of reporting across settings. Furthermore, it is important to understand from the women’s perspective whether a clear informed consent process at the start and information about the study’s purpose are enablers that help them to open up more and report the pregnancy or APO.

### Strengths and limitations

Amongst the strengths of this study is the large, multi-country collection of comprehensive qualitative data from both Africa and Asia, with major efforts made for comparable data collection and analyses to allow comparisons between sites and also between differing APOs. The inclusion of adolescents and women who had suffered APOs allowed for a more diverse picture than previous studies. Additionally, we also held FGDs with data collectors, whose input is often excluded. These were a rich source of information regarding methodological aspects of survey completion, but also in terms of their interaction with respondents, their understanding of local cultures and different field experiences and enablers that are often undocumented. Furthermore, we were able not only to highlight perceptions and experiences of respondents, but to indicate the link between these and reporting of pregnancy and APOs.

Limitations include that although our FGDs were designed to obtain specific information about women’s experiences of the EN-INDEPTH survey, respondents often confused this with routine HDSS data collection. However, this is unlikely to have affected the results as many of the barriers and enablers to reporting were the same. Additionally, the interview guide was sometimes found to be long and repetitive, resulting in shorter FGDs since the facilitators went faster and so did not go into depth and reach saturation about some of the outcomes, especially since some women had their young children with them or needed to return home and complete domestic chores. Furthermore, although termination of pregnancy is among APOs, it is excluded from this paper but focused on in another in this series [47]. Furthermore, the study was done within HDSS sites, whose residents often undergo rounds of interviews, including about pregnancy and APOs. They may therefore be more open to reporting than women residing in non-surveillance populations, which may limit the generalisability of the findings. However, substantial barriers to reporting were found even in this population and barriers are likely to be even greater elsewhere. Finally, women’s report of personal experiences may be different in an FGD with other people around, compared to during an in-depth interview (IDI). Nevertheless, further study to understand reporting especially of APOs will be undertaken as part of a PhD study in one site, using IDIs.

## Conclusions

Our large qualitative study shows that reporting of pregnancies and APOs is influenced by many constructs that are surprisingly consistent across these five LMIC settings. When designing and implementing a survey on pregnancy or APOs, crosscutting issues include careful tool development and translation, informed by a local understanding of terms and words for APOs. The interviewers and their skills remain vital for successful data collection, as does a good understanding of socio-cultural factors and the often unrecognised psycho-social barriers.

Each year, there are approximately 200 million pregnancies globally and our data show that in the settings with the highest fertility rates, these pregnancies are often hidden, putting women and their babies at risk as they do not seek care, contributing to the five million stillbirths and neonatal deaths each year.

Understanding barriers and enablers to reporting these outcomes is important for data improvement, especially in surveys. However, these same barriers also impede societal recognition and attention. We need to ensure that pregnancy and adverse outcomes do not remain hidden and treated as women’s business and that investments in preventing these outcomes and supporting the women who experience them are made.

## Supplementary information

**Additional file 1: **FGD discussion guides.

**Additional file 2: ** COREQ (COnsolidated criteria for Reporting Qualitative research) checklist.

**Additional file 3: ** Background details of the Health and Demographic Surveillance System sites in the EN-INDEPTH study.

**Additional file 4: ** A Socio-demographic details of women in the EN-INDEPTH FGDs. B Socio-demographic details of data collectors in the EN-INDEPTH FGDs.

**Additional file 5: ** Ethical approval of local Institutional Review Boards.

## Data Availability

The datasets generated during the current study are deposited online at 10.17037/DATA.00001556 with data access subject to approval by collaborating parties.

## Abbreviations

APOs: Adverse pregnancy outcomes
CRVS: Civil registration and vital statistics
DHS: Demographic and Health Surveys
ENAP: Every Newborn Action Plan
EN-INDEPTH: Every Newborn - International Network for the Demographic Evaluation of Populations and their Health
FGD(s): Focus group discussion(s)
GBV: Gender-based violence
HDSS: Health and Demographic Surveillance System
IDI: In-depth interview
LMICs: Low- and middle-income countries
MICS: Multiple Indicator Cluster Surveys
SDGs: Sustainable Development Goals
SSA: Sub-Saharan Africa
