# Supplementary material for: Barriers and enablers to reporting pregnancy and adverse pregnancy outcomes in population-based surveys: EN-INDEPTH study
Source: Popul Health Metr. 2021 Feb 8;19(Suppl 1):15. doi: 10.1186/s12963-020-00228-x (PMC7869448; doi:10.1186/s12963-020-00228-x)
Supplement: Supplementary file 1 — Additional file 1: FGD discussion guides. [file 12963_2020_228_MOESM1_ESM.docx]

## Additional file 1: FGD discussion guides

**FOCUS GROUP DISCUSSION GUIDE FOR WOMEN: ENAP_INDEPTH SURVEY**

Interviewer ID **___ ___ ___ ___**

Note taker ID **___ ___ ___ ___**

Interview date: **__ __ / __ __ / ___ ___**

**(***DD/MM/YY***)**

Number of women in the FGD**___ ___ ___ ___**

Location of FGD**___ ___ ___ ___**

**Introduction:**

- Introduce yourself and the note taker
  - Ensure that the informants are comfortable, have time, and are able to participate in the FGD.
- Provide information about the study. Review the purpose of the FGD
  - Explain to the informants what to expect of the FGD
- Explain to the informants that:
- You especially want to learn about their experiences and thoughts on this topic
- The themes that emerge in this group will be put together with those from other groups to help get a wide understanding of what people think about this topic.

*Consent, Anonymity and Recording*

Read the consent form and obtain the participants ‘consent to proceed with the FGD. DO NOT proceed without informed consent. Ask for permission to record the interview. Assure the participants that despite being recorded, you would like to promise them that the discussion will be anonymous. The recordings will be kept safely until they are transcribed word for word, then they will be destroyed. The transcribed notes of the FGD will contain no information that would allow individual subjects to be linked to specific statements. Participants should try to answer as truthfully as possible. Nobody should discuss the comments of other group members once the FGD ends. If there are any questions or discussions that one does not wish to answer or participate in, they do not have to do so. However, they should please try to answer and be as involved as possible.

*Set the ground rules*

- The most important rule is that only one person speaks at a time. There may be a temptation to jump in when someone is talking but please wait until they have finished.
- There are no right or wrong answers
- You do not have to speak in any particular order
- When you have something to say, please do so. There are many of you in the group and it is important that I obtain the views of each of you
- You do not have to agree with the views of other people in the group
- Does anyone have any questions? (answers).
- OK, let’s begin

Start the audio recorder.

Time interview started: : **:**

- Draw a diagram of the seating arrangement.
  - Give each participant a code; Identify the seating of the participants in relation to the facilitator and recorder using the code

**Introduction**

***Start by explaining the difference between the HDSS and the ENAP-INDEPTH survey: The HDSS refers to (explain as per location, name, whom they interview, and frequency of the surveys…..***

***The ENAP-INDEPTH survey was done in the past few months by other people (in some sites by the HDSS), who came and only interviewed women. They were using a tool like the one used by the DHS (the big survey usually done by Government once in a few years)***

**Part A: Experiences with the HDSS data collection process**

Now we are going to briefly discuss the questions which the HDSS asks and the information they collect when their interviewers come to your communities. We would like to find out your thoughts.

1. Sometimes in this community, interviewers from the HDSS come to ask you questions about you and your family. These questions include asking about whether anyone is pregnant and the health of your children. Are you comfortable with questions they ask about your pregnancies and your children?

- **Probe:** What are some of the things you like about the questions they ask? What are some of the things that you are not comfortable with concerning the questions they ask?

**Part B: Experiences with the survey data collection process (ENAP-INDEPTH survey)**

Next, we would like to talk to you about the survey that just ended (ENAP – INDEPTH survey, not HDSS).

1. Some interviews have just been done, where they only talked to women, unlike the usual interviews by the HDSS. What do you think about the just ended interviews that only interviewed women? What did other women in this community think about these interviews?

- **Probe:** Time spent; convenience; questions asked; how male partners related to females being spoken to alone

1. **Additional question:** During the interviews/survey that just ended (that only involved women), they were asked questions in 2 different ways:
2. Some were asked about every time that they had ever been pregnant in their life (even if the pregnancy ended early or the baby was not born alive).
3. Other women were only asked about all their babies that were born alive and then asked separately about any stillbirths or miscarriages in the last 5 years.

In this community, do you think that it is the same from a woman’s point of view, to talk to an interviewer about:

1. Every time that a woman had ever been pregnant in her life (even if the pregnancy ended early or the baby was not born alive), compared to
2. Talking about all her babies that were born alive and then asking separately about any stillbirths or miscarriages in the last 5 years.

What is the benefit of each method? What is the disadvantage of each method? Why?

**Part C: Reporting and disclosure of pregnancy**

In this section, we are going to discuss how women in this community share information about their pregnancy, and how other people around them respond. We would like to find out your thoughts on some questions we have.

1. In this community, when a woman thinks she is pregnant who does she tell first?

- **Probe:** Who else does she tell?

1. When does she tell other people? Why does she wait/ not wait to tell other people in the community? What are the challenges that stop people from talking about pregnancy in this community?

- **Probe:** Social, religious and cultural issues; talking to strangers; what do they answer if directly asked about pregnancy

1. During the interviews/survey that just ended (ENAP-INDEPTH survey that only involved women), did you face any challenges talking to the interviewers about your pregnancies? What made it difficult to talk about this? Did any things make it easier? If yes, please explain which ones.

- **Probe:** Social, religious and cultural barriers; is it easier to talk to a man or woman; is it easier to tell a stranger than people you know?
- **Probe:** What about if the baby is stillborn? Why is it important / why is it not important?

**Part D: Reporting / disclosure of** **adverse pregnancy outcomes (neonatal deaths, stillbirths, miscarriages and / or abortions)**

Next, we are going to discuss a topic that is sad. Sometimes, a woman may be pregnant but unfortunately, the baby dies. The baby may die in the first months of the pregnancy, later on after seven months, or while she is giving birth. The baby can also be born alive, but dies before one month has passed. This may even have happened for some of you. We now want to talk about these deaths. It is a sad topic, but it will later help us to know why the babies die and what we can do to save their lives.

1. In this community, when babies die, do you have a name for this type of death? (probe for neonatal deaths, stillbirths, miscarriages and / or abortions)

Do people talk about it or do they keep quiet? Is it different if a baby is stillborn compared to if it is born alive and then dies? (if so how? /why?)

- **Probe:** Do the mothers talk about it? Whom do they talk to; when? Does the family talk about it to non-family members? (Probe for differences in reporting neonatal deaths, stillbirths, and miscarriages and / or abortions)
- **Probe:** Why do people talk about the deaths or not; what do you think makes it hard to report these deaths among babies? (In the usual HDSS interviews; in the just ended survey; in the community)? What makes it easier for women to talk about it?
- **Probe:** Socio-cultural; legal; religious; economic barriers / enablers; would it be easier or harder to talk to a man or woman; somebody you know or a stranger?
- **Probe:** Do you think it is important to report these deaths to health authorities? Why or why not?

**Part E: Gestational age and Birth weight**

In some cases when a woman is pregnant, babies are born too early or too small. We would like to learn from you a few things about this topic.

1. In this community do people think that it is important to count gestational age (from the first day of the woman's last menstrual cycle to the current date/ date when last pregnancy ended?)

- **Probe:** Why is it important? /Why is it not important? If it is important, is it easy for women to count this in this community? Why/ Why not?

1. In this community, do people think that weighing babies when they are born is important?

- **Probe:** Why is it important/ why is it not important? If it is important, how does a mother find out her baby’s birthweight in this community? Are there any problems with getting a baby weighed?
- **Probe:** What about if the baby is stillborn? Why is it important / why is it not important?

**Part E: Knowledge and practices around adverse pregnancy outcomes (neonatal deaths, stillbirths, miscarriages and/ or abortions)**

1. When a baby dies soon after birth in this community, what happens?

Is it different if a baby dies a few weeks after birth? (if so how?/why?)

What happens when a baby is stillborn? Is it different then? (if so how?/why?)

What about if there is an abortion? Is it different then? (if so how?/why?)

(First ask about the neonatal deaths, then stillbirths, miscarriages and/or abortions). For each outcome, probe as below

- **Probe:** Mourning and burial practices; community reactions; spiritual issues; practices of the family members, practices at the health facility if the baby died there; reaction of the family especially partner and in-laws; counselling for families; other support measures for victims
- **Probe:** Which people make the decision on how to deal with the death? Role of women; cultural leaders; traditional healers; traditional birth attendants; religious leaders; any other key stakeholders

1. In such situations, what happens to the women who lose their pregnancy/baby? Is it different if a baby is stillborn compared to if it is born alive and then dies? (if so how?/why?)

- **Probe:** Response of family; community; blame of the mother and stigma; empathy or support; what about those to whom it happens more than once?
- **Probe:** How do the mothers cope during this time? Role of community, healthcare providers, other organizations

**Part F: Recommendations**

Finally, we would like to conclude our discussion by asking for your advice.

1. In your opinion, what do you think can be done so that during surveys (like ENAP-INDEPTH survey and HDSS rounds), the interviewers can get better information on pregnancies, births and babies who unfortunately die?

- **Probe:** Who do you think are the best people to collect this kind of information? Why?

**Time FGD ended: ___ ___ ___ ___ ___**

**Thank the participant for their time. Remind them that the information will be kept confidential.**

**FOCUS GROUP DISCUSSION FOR FIELDWORKERS: ENAP_INDEPTH SURVEY (FIELDWORKERS IN THE EN-INDEPTH SURVEY)**

Interviewer ID **___ ___ ___ ___**

Note taker ID **___ ___ ___ ___**

Interview date: **__ __ / __ __ / ___ ___**

**(***DD/MM/YY***)**

Number of respondents in the FGD**___ ___ ___ ___**

Location of FGD**___ ___ ___ ___**

**Introduction:**

- Introduce yourself and the note taker
  - Ensure that the informants are comfortable, have time, and are able to participate in the FGD.
- Provide information about the study. Review the purpose of the FGD
  - Explain to the informants what to expect of the FGD
- Explain to the informants that:
- You especially want to learn about their experiences and thoughts on this topic
- The themes that emerge in this group will be put together with those from other groups to help get a wide understanding of what people think about this topic.

*Consent, Anonymity and Recording*

Read the consent form and obtain the participants ‘consent to proceed with the FGD. DO NOT proceed without informed consent. Ask for permission to record the interview. Assure the participants that despite being recorded, you would like to promise them that the discussion will be anonymous. The recordings will be kept safely until they are transcribed word for word, then they will be destroyed. The transcribed notes of the FGD will contain no information that would allow individual subjects to be linked to specific statements. Participants should try to answer as truthfully as possible. Nobody should discuss the comments of other group members once the FGD ends. If there are any questions or discussions that one does not wish to answer or participate in, they do not have to do so. However, they should please try to answer and be as involved as possible.

*Set the ground rules*

- The most important rule is that only one person speaks at a time. There may be a temptation to jump in when someone is talking but please wait until they have finished.
- There are no right or wrong answers
- You do not have to speak in any particular order
- When you have something to say, please do so. There are many of you in the group and it is important that I obtain the views of each of you
- You do not have to agree with the views of other people in the group
- Does anyone have any questions? (answers).
- OK, let’s begin

Start the audio recorder.

Time interview started: : **:**

- Draw a diagram of the seating arrangement.
  - Give each participant a code
  - Identify the seating of the participants in relation to the facilitator and recorder using the code

**Part A: Experiences with the overall survey data collection process**

1. *We would like to begin by talking about your experience with data collection during the recently concluded survey.*
2. What were the community perceptions of the survey? (**Probe**: Were they welcomed; how did the men react? Probe for positive and negative reactions)
3. What were the responses of the women who were interviewed? (**Probe**: Was it easy for women to answer? Were they in a hurry? Probe for positive and negative reactions)
4. What challenges did you face in this survey? How did you overcome them?

**Part B: Experiences with the survey questionnaires– general comparison of birth and pregnancy history**

*Next, we would like to specifically talk about the questionnaires used during the survey that you just completed, and compare the birth history to the pregnancy history questionnaire.*

1. Did you find it easier to administer the birth history or the pregnancy history? Why?

- **Probe**: Advantages and disadvantages of both tools
- **Probe**: What are your thoughts on the time taken to administer the tools? What approach do you think gets more information? Why?

**Part C: Collecting data on pregnancy**

*Now, we would like to specifically talk about your experiences collecting data on pregnancies during the just concluded survey.*

1. Were there any challenges you faced in collecting data on pregnancy? If so, please explain them

- **Probe:** socio-cultural and religious issues; community attitudes; sex of interviewer; type of tool; location; ethnicity

1. What challenges did you face in collecting data on gestational age (counting from the first day of the woman's last menstrual cycle to the current date / date of the last pregnancy)?

- **Probe:** difficulties for women in counting gestational age; why such difficulties

1. What challenges did you face in collecting data on birth weight for the women’s children?

- **Probe:** difficulties for women in knowing the correct birth weight; why such difficulties; availability of health cards

1. Were there things that made it easier to collect information on pregnancy?

- **Probe**: socio-cultural and religious issues; community attitudes; gender; type of tool; location; ethnicity

**Part D: Collecting data on negative / adverse pregnancy outcomes (miscarriages; stillbirths; neonatal deaths; abortions)**

*In this section, we shall specifically discuss your experiences collecting data on negative / adverse pregnancy outcomes during the survey that just ended. (Moderator should emphasise what adverse pregnancy outcomes are).*

1. Were there any challenges you faced in collecting data on negative / adverse pregnancy outcomes? If yes, please explain them

- **Probe:** socio-cultural and religious issues; community attitudes; sex of interviewer; type of tool-birth and pregnancy history; location; ethnicity *(With each question here, remember to probe for the different types of outcomes- first neonatal deaths; stillbirths; and finally if possible miscarriages and abortions)*

1. Were there things that made it easier to collect information on negative / adverse pregnancy outcomes?

- **Probe:** socio-cultural and religious issues; community attitudes; sex of interviewer; type of tool-birth and pregnancy history; location; ethnicity *(With each question here, remember to probe for the different types of outcomes - first neonatal deaths; stillbirths; and finally if possible miscarriages and abortions)*

**Part E: Recommendations**

*As we conclude, we would like to ask for your advice*

1. In your opinion, what do you think can be done so that during surveys the interviewers can get better information on pregnancies, births and babies who unfortunately die?

**Time FGD ended: ___ ___ ___ ___ ___**

**Thank the participant for their time. Remind them that the information will be kept confidential.**
