# Supplementary material for: Barriers and enablers to reporting pregnancy and adverse pregnancy outcomes in population-based surveys: EN-INDEPTH study
Source: Popul Health Metr. 2021 Feb 8;19(Suppl 1):15. doi: 10.1186/s12963-020-00228-x (PMC7869448; doi:10.1186/s12963-020-00228-x)
Supplement: Supplementary file 2 — Additional file 2: COREQ (COnsolidated criteria for Reporting Qualitative research) checklist. [file 12963_2020_228_MOESM2_ESM.docx]

## Additional file 2: COREQ (COnsolidated criteria for Reporting Qualitative research) checklist

| **Topic** | **Item No. Guide Questions/Description** | | **Reported on**  **Page No.** |
| --- | --- | --- | --- |
| **Domain 1: Research team**  **and reﬂexivity** | | | |
|  |  |  |  |
| *Personal characteristics* | | | |
| Interviewer/facilitator | 1 Which author/s conducted the interview or focus group? | |  |
| Credentials | 2 What were the researcher’s credentials? E.g. PhD, MD | |  |
| Occupation | 3 What was their occupation at the time of the study? | |  |
| Gender | 4 Was the researcher male or female? | |  |
| Experience and training | 5 What experience or training did the researcher have? | |  |
| *Relationship with*  *participants* | | | |
|  |  |  |  |
| Relationship established | 6 Was a relationship established prior to study commencement? | |  |
| Participant knowledge of | 7 What did the participants know about the researcher? e.g. personal  goals, reasons for doing the research | |  |
| the interviewer |  |  |  |
| Interviewer characteristics | 8 What characteristics were reported about the inter viewer/facilitator?  e.g. Bias, assumptions, reasons and interests in the research topic | |  |
| **Domain 2: Study design** | | | |
| *Theoretical framework* | | | |
| Methodological orientation | 9 What methodological orientation was stated to underpin the study? e.g.  grounded theory, discourse analysis, ethnography, phenomenology, content analysis | |  |
| and Theory |  |  |  |
| *Participant selection* | | | |
| Sampling | 10 How were participants selected? e.g. purposive, convenience,  consecutive, snowball | |  |
| Method of approach | 11 How were participants approached? e.g. face-to-face, telephone, mail,  email | |  |
| Sample size | 12 How many participants were in the study? | |  |
| Non-participation | 13 How many people refused to participate or dropped out? Reasons? | |  |
| *Setting* | | | |
| Setting of data collection | 14 Where was the data collected? e.g. home, clinic, workplace | |  |
| Presence of non- | 15 Was anyone else present besides the participants and researchers? | |  |
| participants |  |  |  |
| Description of sample | 16 What are the important characteristics of the sample? e.g. demographic  data, date | |  |
| *Data collection* | | | |
| Interview guide 17 | | Were questions, prompts, guides provided by the authors? Was it pilot  tested? |  |
| Repeat interviews 18 | | Were repeat inter views carried out? If yes, how many? |  |
| Audio/visual recording 19 | | Did the research use audio or visual recording to collect the data? |  |
| Field notes 20 | | Were ﬁeld notes made during and/or after the inter view or focus group? |  |
| Duration 21 | | What was the duration of the inter views or focus group? |  |
| Data saturation 22 | | Was data saturation discussed? |  |
| Transcripts returned 23 | | Were transcripts returned to participants for comment and/or |  |
|  | |  |  |

Developed from: Tong A, Sainsbury P, Craig J. Consolidated criteria for reporting qualitative research (COREQ): a 32-item checklist for interviews and focus groups. *International Journal for Quality in Health Care*. 2007. Volume 19, Number 6: pp. 349 – 357
