# Supplementary material for: Barriers and enablers to reporting pregnancy and adverse pregnancy outcomes in population-based surveys: EN-INDEPTH study
Source: Popul Health Metr. 2021 Feb 8;19(Suppl 1):15. doi: 10.1186/s12963-020-00228-x (PMC7869448; doi:10.1186/s12963-020-00228-x)
Supplement: Supplementary file 3 — Additional file 3: Background details of the Health and Demographic Surveillance System sites in the EN-INDEPTH study. [file 12963_2020_228_MOESM3_ESM.docx]

## Additional file 3: Background details of the Health and Demographic Surveillance System sites in the EN-INDEPTH study (n=5)

|  | **Bandim (Guinea-Bissau)** | **Dabat (Ethiopia)** | **IgangaMayuge (Uganda)** | **Kintampo (Ghana)** | **Matlab (Bangladesh)** |
| --- | --- | --- | --- | --- | --- |
| **Country** | Guinea-Bissau | Ethiopia | Uganda | Ghana | Bangladesh |
| **Residence** | Rural and urban | Rural | Rural | Rural | Rural |
| **Population** | 180,000 | 69,468 | 83,000 | 152,519 | 230,185 |
| **Livebirths (per year)** | 5,790 | 1320 | 2264 | 4710 | 4863 |
| **Stillbirths (per year)** | 297 | 34 | 45 | 86 | 92 |
| **Stillbirth rate, per 1000 total births (95%CI)** | 49 (43.6-54.9) | 25 (17.3 – 34.9) | 19 (14.7 – 24.2) | 18 (14.4 – 22.3) | 19 (15.3 – 23.2) |
| **Neonatal deaths (per year)** | 204 | 53 | 118 | 95 | 104 |
| **Neonatal mortality rate, per 1000 total births (95%CI)** | 35 (30.5 – 40.1) | 40 (30.2 – 52.2) | 52 (43.3 – 62.1) | 20 (16.2 – 24.4) | 21 (17.1 – 25.4) |
