# Supplementary material for: Barriers and enablers to reporting pregnancy and adverse pregnancy outcomes in population-based surveys: EN-INDEPTH study
Source: Popul Health Metr. 2021 Feb 8;19(Suppl 1):15. doi: 10.1186/s12963-020-00228-x (PMC7869448; doi:10.1186/s12963-020-00228-x)
Supplement: Supplementary file 4 — Additional file 4: A Socio-demographic details of women in the EN-INDEPTH FGDs. B Socio-demographic details of data collectors in the EN-INDEPTH FGDs. [file 12963_2020_228_MOESM4_ESM.docx]

## Additional file 4A: Socio-demographic details of women in the EN-INDEPTH FGDs (n=172)

| **Characteristic** | **Bandim (Guinea-Bissau)** | **Dabat (Ethiopia)** | **IgangaMayuge (Uganda)** | **Kintampo (Ghana)** | **Matlab (Bangladesh)** |
| --- | --- | --- | --- | --- | --- |
| **Total number of FGDs** | 4 | 3 | 4 | 4 | 4 |
| **Total number of respondents** | 29 | 25 | 40 | 50 | 28 |
| **Age (years)**  <25  25-35  35-49  50+  Missing | 8  15  6  0  0 | 0  13  11  0  1 | 15  11  14  0  0 | 14  25  10  0  1 | 13  3  12  0  0 |
| **Religion**  Christian  Muslim  Other or none | 41.0%  39.4%  19.5% | 95.9%  4.1%  0.0% | 45.8%  54.1%  0.2% | 63.1%  30.8%  6.1% | 0.0%  88.5%  11.5% |
| **Ethnicity** | 22.3% Pepel  21.0% Fula  11.7% Balante  10.3% Manjaco  34.7% Others | 99.8% Amhara  0.07% Tigray  0.04% Oromo | 84.6% Basoga  15.4% Others | 20.5% Akan  14.7% Dagarti  12.5% Konkomba  10.5% Mo  8.2% Gonja  33.6% Others | 99.3% Bengali  0.02% Chakma  0.7% Others |
| **Study language** | Creole (Balanta in a few cases) | Amharic | Lusoga | Twi | Bengali |

## Additional file 4B: Socio-demographic details of data collectors in the EN-INDEPTH FGDs (n=82)

| **Characteristic** | **Bandim** | **Dabat** | **IgangaMayuge** | **Kintampo** | **Matlab** |
| --- | --- | --- | --- | --- | --- |
| **Total number of FGDs** | 1 | 1 | 2 | 2 | 3 |
| **Total number of respondents** | 11 | 10 | 20 | 18 | 23  (8 of these were supervisors) |
| **Sex**  Male  Female | 2  9 | 3  7 | 10  10 | 15  3 | 4 (supervisors)  19 (15 data collectors, 4 supervisors) |
| **Age (years)**  <25  25-35  35-49  50+  Missing | 6  3  2  0  0 | 8  2  0  0  0 | 0  10  10  0  0 | 1  13  4  0  0 | 2  7  13  1  0 |
| **Study language** | Creole (Balanta in a few cases) | Amharic | Lusoga and English | Twi and English | Bengali |
