# Supplementary material for: Barriers and enablers to reporting pregnancy and adverse pregnancy outcomes in population-based surveys: EN-INDEPTH study
Source: Popul Health Metr. 2021 Feb 8;19(Suppl 1):15. doi: 10.1186/s12963-020-00228-x (PMC7869448; doi:10.1186/s12963-020-00228-x)
Supplement: Supplementary file 5 — Additional file 5: Ethical approval of local Institutional Review Boards. [file 12963_2020_228_MOESM5_ESM.docx]

## Additional file 5: Ethical approval of local Institutional Review Boards

| **Site** | **Institutional Review Boards** | **Date** | **Number/Ref** |
| --- | --- | --- | --- |
| Bandim (Guinea-Bissau) | Comité Nacional de Ética na Saúde | 12 June 2017 | 072/CNES/INASA/2017 |
| Dabat (Ethiopia) | Institutional Review Board, University of Gondar | 19 April 2017 | VP/RCS/05/1074/2016 |
| IgangaMayuge (Uganda) | Mildmay Uganda Research Ethics Committee  Uganda National Council of Science and Technology | 26 June 2017  11 October 2017 | REC REF 0305-2017  SS 4244 |
| Kintampo (Ghana) | Kintampo Health Research Centre, Ghana Health service Scientific Review Committee (SRC)  Ghana Health Services Ethics Review Committee  Kintampo Health Research Centre Institutional Ethics Committee | 14 June 2017  26 July 2017  9 August 2017 | SRC/130617  GHS-ERC:19/06/14  KHRCIEC/2017-14 |
| Matlab (Bangladesh) | Icddr,b Ethical Review Committee | 19 July 2017 | PR-17049 |
| London School of Hygiene & Tropical Medicine (United Kingdom) | London School of Hygiene & Tropical Medicine | 24 May 2017 | 12218 |
